# Enzyme-modified indium tin oxide microelectrode array-based electrochemical uric acid biosensor

**DOI:** 10.1186/2194-0517-2-5

**Published:** 2013-02-22

**Authors:** Nidhi Puri, Vikash Sharma, Vinod K Tanwar, Nahar Singh, Ashok M Biradar, Rajesh

**Affiliations:** Polymer and Soft Material Section, CSIR-National Physical Laboratory, Dr. K.S. Krishnan Road, New Delhi, 110012 India

**Keywords:** Uric acid, Self-assembled monolayer, Microfluidic, PDMS, Amperometric sensor

## Abstract

We fabricated a miniaturized electrochemical uric acid biosensor with a 3-aminopropyltriethoxysilane (APTES)-modified indium tin oxide (ITO) microelectrode array (μEA). The ITO-μEA on a glass plate was immobilized with the enzyme uricase, through a cross-linker, bis[sulfosuccinimidyl]suberate (BS^3^). The enzyme-immobilized electrode (uricase/BS^3^/APTES/ITO-μEA/glass) was characterized by atomic force microscopy and electrochemical techniques. The cyclic voltammetry and impedance studies show an effective binding of uricase at the μEA surface. The amperometric response of the modified electrode was measured towards uric acid concentration in aqueous solution (pH 7.4), under microfluidic channel made of polydimethylsiloxane. The μEA biosensor shows a linear response over a concentration range of 0.058 to 0.71 mM with a sensitivity of 46.26 μA mM^−1^ cm^−2^. A response time of 40 s reaching a 95% steady-state current value was obtained. The biosensor retains about 85% of enzyme activity for about 6 weeks. The biosensor using μEA instead of a large single band of electrode allows the entire core of the channel to be probed though keeping an improved sensitivity with a small volume of sample and reagents.

**Electronic supplementary material:**

The online version of this article (doi:10.1186/2194-0517-2-5) contains supplementary material, which is available to authorized users.

## Background

Lab-on-a-chip has become a very popular concept since its inception about a decade ago. It possesses many remarkable features which include the ability to fully integrate all preparation, detection, and analytical processes into a single chip no bigger than the size of a microscopic slide, high throughput, short analysis time, small volume, and high sensitivity ([Lee and Lee 2004]). When compared to their macrocounterparts, scaling down of electrochemical systems via a microelectrode array (μEA) is anticipated to make the sample size as well as concentration smaller and the electron transfer faster ([Wang et al. 2009]). Measurement of the concentration of species relies on the chemical phenomena that involve charge transfer (like redox reactions) from or to the electrode. The amount of charge transferred is a direct signal of the concentration of the species, and this can be measured as the charge itself (coulometry), as a current ([amperometry; Wang et al. 2009][; Quintino et al. 2005]), or as a voltage ([potentiometry; Wilson and Dewald 2001][; Muñoz and Palmero 2005]).

A variety of methods for fabrication of microfluidic devices with subsequent bonding to form channels include photolithography-lift-off ([Schoning et al. 2005]), soft lithography ([Kwakye and Baeumner 2003]), and laser ablation ([Eswara and Dutt 1974]). Whitesides et al. ([2001]) has reviewed the relevance of soft lithography for the fabrication of microchannels which is faster, cheaper, and more suitable for most biological applications. In device fabrication, the surface alteration and fluid management within the chip are mainly controlled by the surface property of the material, while the detection modem is governed by its optical property ([Henaresa et al. 2008]).

Uric acid (UA), a final outcome of purine metabolism in biological systems, is an important biological molecule present in body fluids, such as blood and urine. Its level can be used as a marker for the detection of disorders associated with purine metabolism and can reveal the status of immunity ([Eswara and Dutt 1974]). Its normal level in serum is between 0.13 and 0.46 mM (2.18 to 7.7 mg dL^−1^[; Raj and Ohsaka 2003]) and in urinary excretion is between 1.49 and 4.46 mM (25 to 74 mg dL^−1^; Matos et al. [2000a], [b]). The presence of abnormal UA levels leads to gout, chronic renal disease, some organic acidemias, leukemia, pneumonia, and Lesch-Nyhan syndrome ([Burtic and Ashwood 1994]).

In this study, we describe an indium tin oxide microelectrode array (ITO-μEA) printed over a glass plate for the quantitative detection of uric acid in aqueous solution integrated into a polydimethylsiloxane (PDMS)-made microfluidic channel. The surface of ITO-μEA was modified with a self-assembled monolayer (SAM) of 3-aminopropyltriethoxysilane (APTES), which was immobilized with the enzyme uricase through a cross-linker, bis[sulfosuccinimidyl]suberate (BS^3^), by forming a strong amide bonding at both ends with free available amino groups of APTES and uricase. The modified electrode (uricase/BS^3^/APTES/ITO-μEA/glass) was characterized by atomic force microscopy (AFM), cyclic voltammetry (CV), and electrochemical impedance spectroscopy (EIS) in the presence of [Fe(CN)_6_]^3−^ as a redox probe.

## Results and discussion

**Figure 1:**
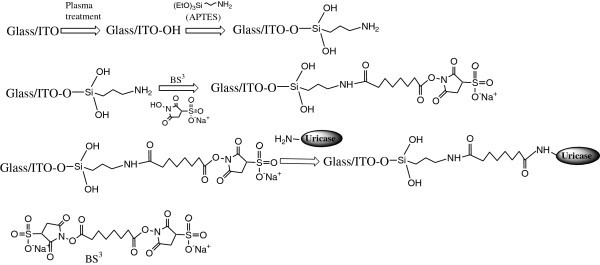
**Schematic illustration of each step of surface modification of ITO-μEA/glass and uricase immobilization.**

**Figure 2:**
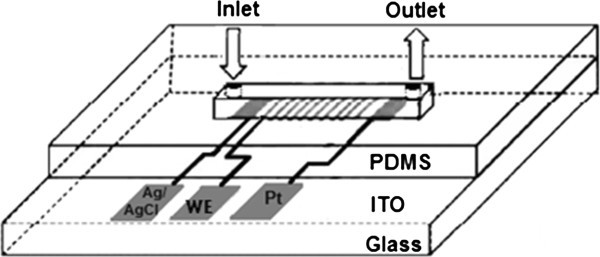
**Schematic diagram of biosensor device with an integrated uricase/BS** ^**3**^ **/APTES/ITO-μEA/glass electrode within a microfluidic channel.**

Figure 1 depicts a scheme for the fabrication of uricase/BS^3^/APTES/ITO-μEA/glass, wherein the free NH_2_ groups present at the surface of the APTES/ITO-μEA/glass electrode have been utilized for the covalent immobilization of uricase through a cross-linking reagent, BS^3^. Figure 2 shows the experimental arrangement of μEA for the detection of uric acid.

### Surface morphology

**Figure 3:**
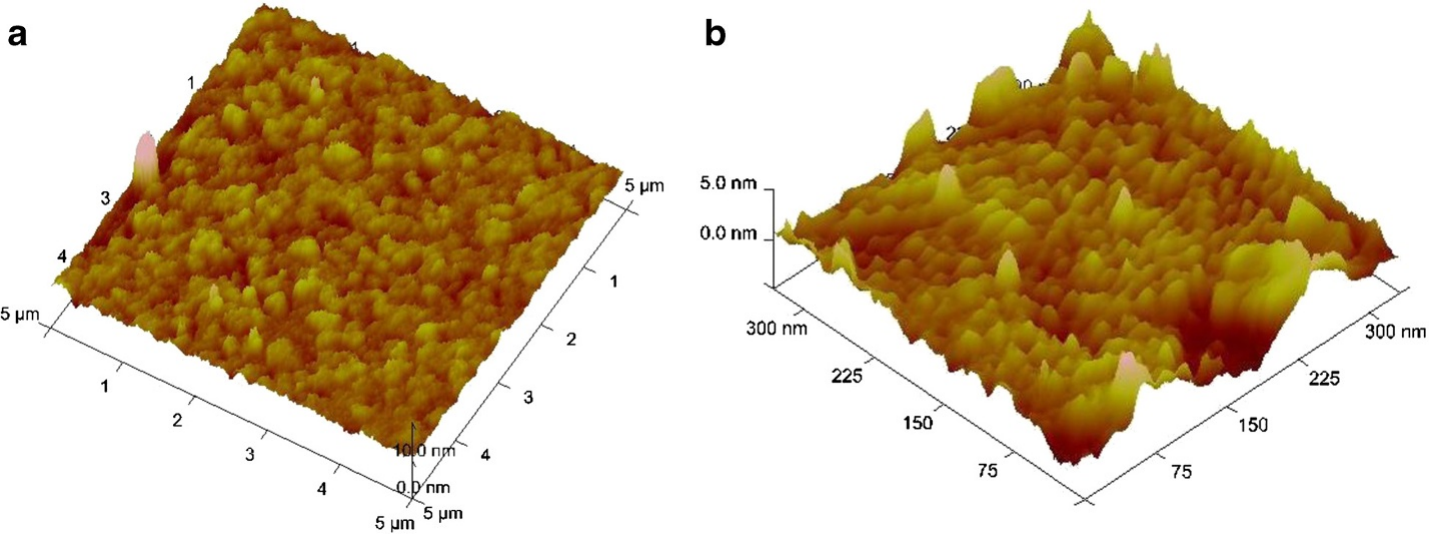
**Contact-mode AFM images of (a) APTES/ITO-μEA/glass and (b) uricase/BS** ^**3**^ **/APTES/ITO-μEA/glass.**

The surface characterization of the biosensor was carried out by taking the AFM images of the modified electrode before and after the enzyme immobilization. The AFM image of the APTES/ITO-μEA/glass (Figure 3a) shows densely arrayed APTES molecules with an average image height of 8.6 nm, which is more than the average thickness of APTES monolayer of 3 to 5 nm, indicating a strong polarity in the amino end group of APTES molecule resulting in increased inclined angle of the APTES molecule chain. This makes the APTES molecule chain more disordered and get piled up easily ([Wang et al. 2005]). The AFM image of uricase-immobilized APTES/ITO-μEA/glass (Figure 3b) exhibits a regular island-like structure. Since the lateral size of the visible image depends upon the convolution effect that arises between the sample and the AFM tip, an increase of about 8 nm in height in the AFM image was found with the uricase-modified APTES/ITO-μEA/glass surface. This increase in the height of the image is in accordance with the size of the uricase molecule ([Akgöl et al. 2008]).

### Electrochemical characterization of uricase/BS^3^/APTES/ITO-μEA/glass electrode

**Figure 4:**
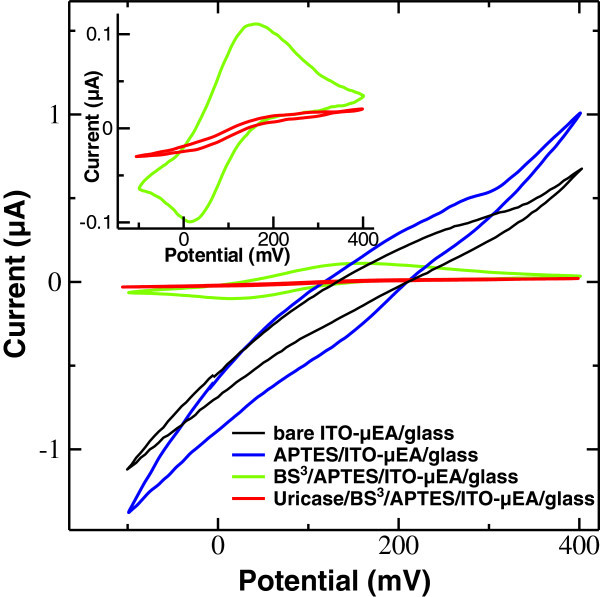
**Cyclic voltammograms of bare ITO-μEA/glass, APTES/ITO-μEA/glass, BS**^**3**^**/APTES/ITO-μEA/glass, and uricase/BS**^**3**^**/APTES/ITO-** **μEA/glass.** In 0.1 M KCl solution containing 2 mM [Fe(CN)_6_]^3−^. The scan rate is 25 mV s^−1^. The third cycle voltammogram is shown. The inset shows enlarged CVs of BS^3^/APTES/ITO-μEA/glass and uricase/BS^3^/APTES/ITO-μEA/glass.

The uricase/BS^3^/APTES/ITO-μEA/glass was characterized by cyclic voltammetry and electrochemical impedance spectroscopy. All electrochemical measurements were performed in phosphate-buffered saline (PBS) solution (pH 7.4) containing 0.1 M KCl and 2 mM [Fe(CN)_6_]^3−^ under a PDMS-made microchannel. Figure 4 shows cyclic voltammograms of the modified electrode before and after the enzyme immobilization. The inset shows a magnified view of CVs that correspond to BS^3^/APTES/ITO-μEA/glass and uricase/BS^3^/APTES/ITO-μEA/glass electrode.

In all CV experiments, the third cycle was considered as a stable one since no significant changes were observed in the subsequent cycles. The bare ITO-μEA/glass shows a quasi-reversible cyclic voltammogram with a peak-to-peak separation between the oxidation and reduction potential (Δ*E*_p_) of 166.51 mV. Upon modification with SAM of APTES, it shows a more reversible signal with decreased Δ*E*_p_ of 144.86 mV between the oxidation and reduction peaks and increased oxidation and reduction current of the redox probe. This is attributed to an increased interfacial concentration of the anionic probe [Fe(CN)_6_]^3−^ due to its strong affinity towards the polycationic (NH_2_) layer ([Zhao et al. 2005]). A further modification with a cross-linker, bis[sulfosuccinimidyl]suberate, results in a significant increase in Δ*E*_p_ of 152.50 mV with a reduction in the redox current due to a repulsive interaction of polyanions (SO_3_−) with the anionic probe [Fe(CN)_6_]^3−^, at the surface interface, confirming the formation of a cross-linker, BS^3^, layer over the surface of APTES/ITO-μEA/glass. This trend of CV curve with an increased Δ*E*_p_ of 195.32 mV and a decreased redox current was further observed after the immobilization of enzyme molecules at the surface of the modified electrode (BS^3^/APTES/ITO-μEA/glass). This indicates an efficient covalent bonding of insulating enzyme molecules to APTES/ITO-μEA/glass through the cross-linker BS^3^, which perturbs the interfacial electron transfer considerably, at the electrode surface.

**Figure 5:**
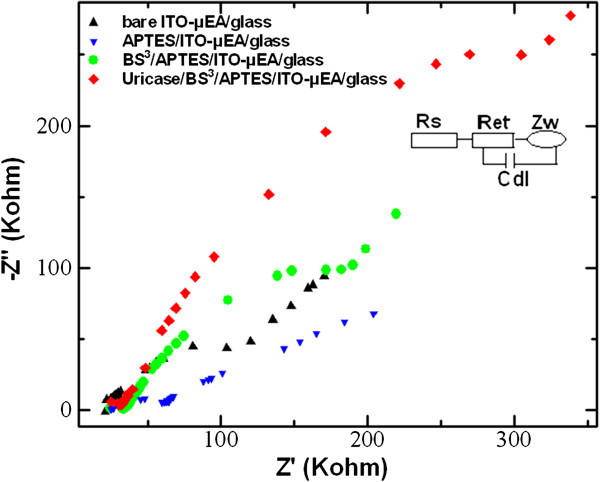
**Nyquist plot obtained for bare ITO-μEA/glass, APTES/ITO-μEA/glass, BS**^**3**^**/APTES/ITO-μEA/glass, and uricase/BS**^**3**^**/APTES/ITO-μEA/glass.** In 0.1 M KCl solution containing 2 mM [Fe(CN)_6_]^3−^.

The electrochemical behavior of the modified electrode was characterized by EIS, using an AC signal of 5-mV amplitude, at a formal potential of the redox couple, at a frequency range of 1 to 100,000 Hz. The Nyquist plot, given in Figure 5, shows the impedance spectra taken in each step of the surface modification. The Nyquist plot was fitted using Randles equivalent circuit, as shown in the inset of Figure 5, and a computer software evaluated EIS parameters. The impedance spectrum showed a semicircle region over a high-frequency range and a linear part at low frequencies, the radius of which corresponds to the charge transfer resistance (*R*_et_). The impedance result is modeled by an electronic equivalent circuit, shown in the inset, for the solution resistance (Rs), the Warburg impedance (Zw), which resulted from the diffusion of ions in a bulk electrolyte, the double layer capacitance (Cdl), and the *R*_et_ for the electrochemical reaction ([Randles 1947]). Figure 5 shows the electrochemical impedance spectra of the bare and the modified ITO-μEA/glass before and after the immobilization of the enzyme uricase, and the corresponding electron transfer resistance values are listed in Table 1. The bare ITO-μEA/glass shows an *R*_et_ value of 2.11 KΩ cm^2^ which decreased to 1.10 KΩ cm^2^ upon treatment with APTES, indicating an easy electronic transport at the electrode surface interface. However, further treatment with a cross-linker, BS^3^, and on subsequent immobilization with the enzyme uricase result in increased *R*_et_ values of 2.90 and 5.17 KΩ cm^2^, respectively. The results obtained in EIS are in well agreement with the trend observed in CV measurements, which further confirms the formation of the uricase/BS^3^/APTES/ITO-μEA/glass.

**Table 1:** **Δ** ***E***_**p**_ **and** ***R***_**et**_ **of biosensor before and after ITO-μEA surface modifications and enzyme immobilization**

	Δ***E***_p_	***R***_et_	***R***_et_
	(mV)	(KΩ)	(KΩ cm^2^)^a^
Bare/ITO-μEA/glass	166.51	145.82	2.11
APTES/ITO-μEA/glass	144.86	76.23	1.10
BS^3^/APTES/ITO-μEA/glass	152.50	200.13	2.90
Uricase/BS^3^/APTES/ITO-μEA/glass	195.32	356.79	5.17

^a^Surface area of ITO-μEA is 1.45 mm^2^; Δ*E*_p_, change in peak-to-peak separation in redox potential; *R*_et_, charge transfer resistance.

### Amperometric response of uricase/BS^3^/APTES/ITO-μEA/glass

**Figure 6:**
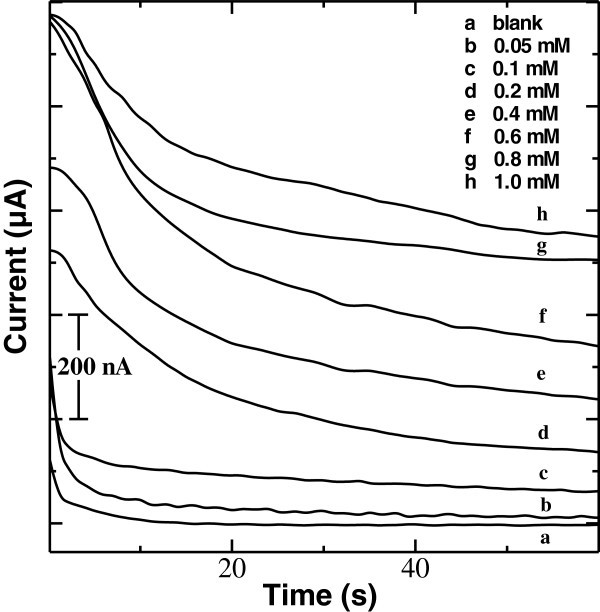
**Chronoamperometric response curve of uricase/BS** ^**3**^ **/APTES/ITO-μEA/glass with different concentrations of uric acid.**

Chronoamperometric response study was carried out with uricase/BS^3^/APTES/ITO-μEA/glass as the working electrode at a bias voltage of 0.26 V vs. Ag/AgCl in PBS (pH 7.4) containing 2 mM [Fe(CN)_6_]^3−^ as the redox mediator. Figure 6 shows a chronoamperometric response of the uricase/BS^3^/APTES/ITO-μEA/glass electrode as a function of uric acid concentration in PBS, in the presence of a redox mediator. An increasing order of amperometric response was observed with increasing uric acid concentration, and 95% steady-state current response to uric acid was obtained in about 40 s.

**Figure 7:**
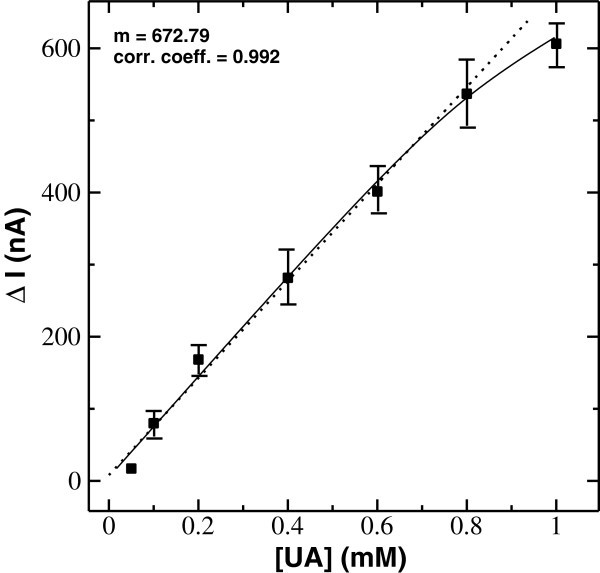
**Steady-state current dependence calibration curve of uricase/BS** ^**3**^ **/APTES/ITO-μEA/glass biosensor to uric acid.**

Figure 7 shows the steady-state current dependence calibration curve to uric acid concentration. The amperometric response of the device to uric acid concentration was found to be linear in the range of 0.058 to 0.71 mM with a correlation coefficient of 0.992 (*n* = 5). The lowest detection limit of the electrode was 0.0084 mM at a signal-to-noise ratio of 3. The sensitivity of the enzyme electrode was calculated from the slope (*m*) of the linearity curve, and it was found to be 46.26 μA mM^−1^ cm^−2^. The stability of the biosensor was studied, under the storage condition of 4°C to 5°C, by continuously monitoring the current response for 0.6 mM uric acid, at an interval of 1 week. The biosensor retained its current response to uric acid with a slow decrement of about 12% over a period of 6 weeks. The biosensor shows a sharp decrement up to about 40% to its initial activity on the basis of amperometric current response that might be due to the limited stability of the enzyme over the silane matrix over a period of time.

## Conclusions

A biosensor was fabricated by immobilizing the enzyme uricase on SAM of APTES via the cross-linker BS^3^ on an ITO-μEA/glass plate. The uricase/BS^3^/APTES/ITO-μEA/glass electrode was characterized by electrochemical techniques and AFM, whereas the amperometric response was studied as a function of uric acid concentration. The biosensor with uricase/BS^3^/APTES/ITO-μEA/glass electrode showed a linear range of 0.058 to 0.71 mM with a lower detection limit of 8.4 μM. The response time was found to be 40 s reaching a 95% steady-state current value. The efficient bonding of the enzyme on the electrode surface exhibits an improved sensitivity of 46.26 μA mM^−1^ cm^−2^. The microfluidic channel provided the controlled volume of the sample to be tested in close proximity to the uricase/BS^3^/APTES/ITO-μEA/glass electrode for a fast electrochemical reaction, wherein the μEA helped in moving the electron through interconnected microelectrode bands having a comparatively reduced area to a single-band-like structure. The easy method of fabrication and the small sample volume requirement together with the high sensitivity towards uric acid measurement make this biosensor advantageous over the recently reported uric acid biosensors (Table 2). This system may be optimized further for selectivity with different interferants using a real blood sample or serum for other species of biomedical importance.

**Table 2:** **Characteristics of some recently reported amperometric uric acid biosensors**

Matrix	Response time (s)	Stability	Linear range	Sensitivity	Bias potential (V)	Reference
SAM of heteroaromatic thiol/Au	-	1 day	1 to 300 μM	0.0149 ± 0.05 μA mM^−1^	~0.4	Raj and Ohsaka ([2003])
*o*-Aminophenol-aniline copolymer	-	~50 days	0.0001 to 0.5 mmol dm^−3^	~2.65 μA mM^−1^	0.4	Pan et al. ([2006])
Ir-C	41	-	0.1 to 0.8 mM	16.60 μA mM^−1^	0.25	Luo et al. ([2006])
Polyaniline	-	~60 days	0.0036 to 1.0 mmol dm^−3^	~1 μA mM^−1^	0.4	Kan et al. ([2004])
Polyaniline-PPy	70	4 weeks	2.5 × 10^-6^ to 8.5 ×10^-5^ M	1.12 μA mM^−1^	0.4	Arslan ([2008])
Poly(allylamine)/poly(vinyl sulfate)			10^−6^ to 10^−3^ M	<5 μA mM^−1^	0.6	Hoshi et al. ([2003])
Polyaniline			0.001 to 1.0 mM dm^−3^	<7 μA mM^−1^	0.4	Jiang et al. ([2007])
ZnS quantum dots		20 days	5.0 × 10^−6^ to 2.0 × 10^−3^ mol L^−1^	2.2 μA mM^−1^	0.45	Zhang et al. ([2006])
Carbon paste	70	>100 days	Up to100 μmol dm^−3^		0.34	Dutra et al. ([2005])
Polypropylene			4.82 to 10.94 mg dL^−1^	0.0029 μA mM^−1^		Chen et al. ([2005])
SAM of PET and DTB on Au	80 to 100		5 to 150 μM	3.4 ± 0.08 nA cm^−2^ μM^−1^	−0.1	Behera and Raj ([2007])
ZnO nanorods			5.0 × 10^−6^ to 1.0 × 10^−3^ mol L^−1^			Zhang et al. ([2004])
GNP-based uric acid biosensor	25		0.07 to 0.63 mM	19.27 μA mM^−1^		Ahuja et al. ([2011])
APTES-based ITO-μEA/glass electrode	40	6 weeks	0.058 to 0.71 mM	46.26 μA mM^−1^ cm^−2^	0.26	Present work

## Methods

### Materials

The enzyme uricase (EC 1.7.3.3, 9 units mg^−1^ from *Bacillus fastidiosus*) was procured from Sigma-Aldrich Corp. (St. Louis, MO, USA). APTES was purchased from Merck Chemicals (Darmstadt, Germany). BS^3^ was obtained from Pierce Biotechnology (Rockford, IL, USA). Uric acid with 99% purity was purchased from CDH (New Delhi, India). Positive photoresist 1300–31 was purchased from Shipley (Marlborough, MA, USA), and negative photoresist SU-8-2025 is from MicroChem Corp. (Newton, MA, USA). Sylgard 184 PDMS was purchased from Dow Corning (Midland, MI, USA). Other chemicals were of analytical grade and used without further purification.

### Apparatus

AFM images were obtained on a VEECO/diCP2 scanning probe microscope (Plainview, NY, USA). CV and EIS measurements were done on a PGSTAT302N AUTOLAB instrument from Eco Chemie (Utrecht, The Netherlands). Impedance measurements were performed in the presence of a redox probe, [Fe(CN)_6_]^3−^, at scanning frequencies from 1 to 100,000 Hz. All measurements were carried out on a three-electrode system with uricase/BS^3^/APTES/ITO-μEA/glass as the working electrode, Ag/AgCl as the reference electrode, and platinum as the counter electrode.

### Fabrication of uricase/BS^3^/APTES/ITO-μEA/glass-embedded microfluidic device

The ITO-coated glass plates (2 × 3 cm^2^) with a typical resistance of approximately 40 Ω were cleaned by ultrasonic cleaning, in chronological order, in soapy water (Extran, Merck Millipore, Billerica, MA, USA), acetone, ethanol, isopropyl alcohol, and double-distilled (DD) water for 10 min each, and drying in vacuum. These cleaned ITO glass plates were then exposed to oxygen plasma in a plasma chamber for 5 min. SAM of APTES was prepared over an ITO glass plate by immersing it in 2% ethanolic solution of APTES for 1.5 h, under ambient conditions. The glass plate was then rinsed with ethanol in order to remove the majority of non-bonded APTES from the surface of the substrate and dried it under N_2_.

A three-electrode system with a pattern of μEA (1.45 mm^2^) as the working electrode and Ag/AgCl and Pt as the reference and counter electrodes, respectively, was printed over a glass plate. The μEA pattern was transferred over an APTES-coated ITO-glass by photolithography using Shipley positive photoresist 1300–31, followed by etching of remaining exposed ITO coating by treating with a suspension of zinc dust and dilute HCl. The ITO-μEA is composed of 42 interconnected microbands having a band width of 65 μm and a band length of 225 μm with an interspacing bandgap of 65 μm, prepared according to a procedure reported earlier ([Chen and White 2011]). The Ag pattern was deposited over the glass plate by e-beam evaporation technique, followed by treatment with 1 mM solution of FeCl_3_ for 10 s, and washed with DD water to obtain an Ag/AgCl reference electrode ([Polk et al. 2006]). The APTES/ITO-μEA/glass electrode was then modified with SAM of BS^3^ by treating it with 5 mM BS^3^ solution prepared in sodium acetate buffer (pH 5.0) for 1.0 h, washed with DD water, and dried under N_2_. The BS^3^-treated APTES/ITO-μEA/glass electrode (BS^3^/APTES/ITO-μEA/glass) was immersed in PBS solution containing approximately 3 U of uricase (pH 7.4) for a period of 1.5 h. The enzyme electrode so formed was washed thrice with PBS (pH 7.4) to remove the excess unbound enzyme and was finally dried under N_2_ at room temperature and stored at 4°C.

A PDMS microfluidic channel was prepared to deliver a controlled sample volume over a glass plate comprising a three-electrode system with a pattern of a uricase/BS^3^/APTES/ITO-μEA/glass working electrode, Ag/AgCl reference electrode, and Pt counter electrode for response measurement. A master was created with Shipley negative photoresist SU-8-2025 on a smooth glass plate by spin coating with a speed of 1,000 rpm and exposing through a photomask under highly intense UV light for 20 s. The resulting master structure was used as a mold to create PDMS blocks equipped with microfluidic channels of 75 μm in height and 0.5 mm × 16 mm area with sample inlet and outlet ports (*D* = 1 mm) punched at the two ends of the microchannel for fluid flow.

## Authors’ original submitted files for images

Below are the links to the authors’ original submitted files for images. Authors’ original file for figure 1 Authors’ original file for figure 2 Authors’ original file for figure 3 Authors’ original file for figure 4 Authors’ original file for figure 5 Authors’ original file for figure 6 Authors’ original file for figure 7
