# Supplementary figures and images for: Enzyme-modified indium tin oxide microelectrode array-based electrochemical uric acid biosensor
Source: Prog Biomater. 2013 Feb 22;2:5. doi: 10.1186/2194-0517-2-5 (PMC5151101; doi:10.1186/2194-0517-2-5)

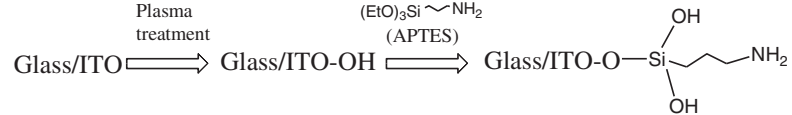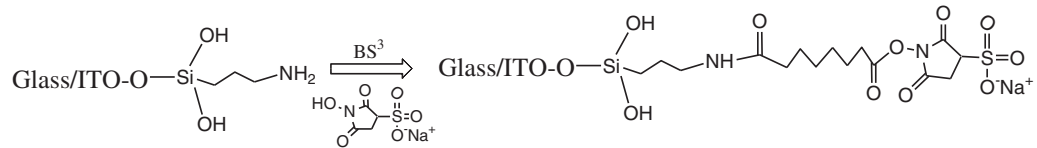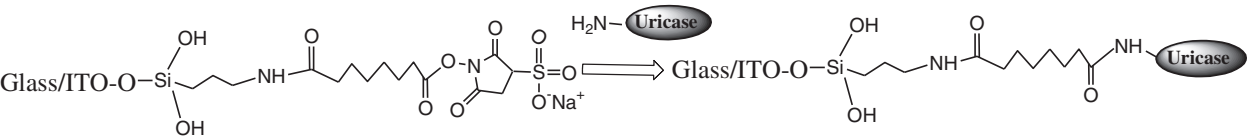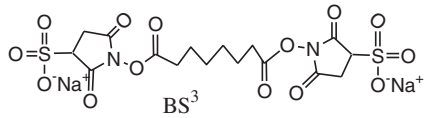

Supplement: Supplementary file 1 — Authors’ original file for figure 1 [file 40204_2012_8_MOESM1_ESM.pdf]

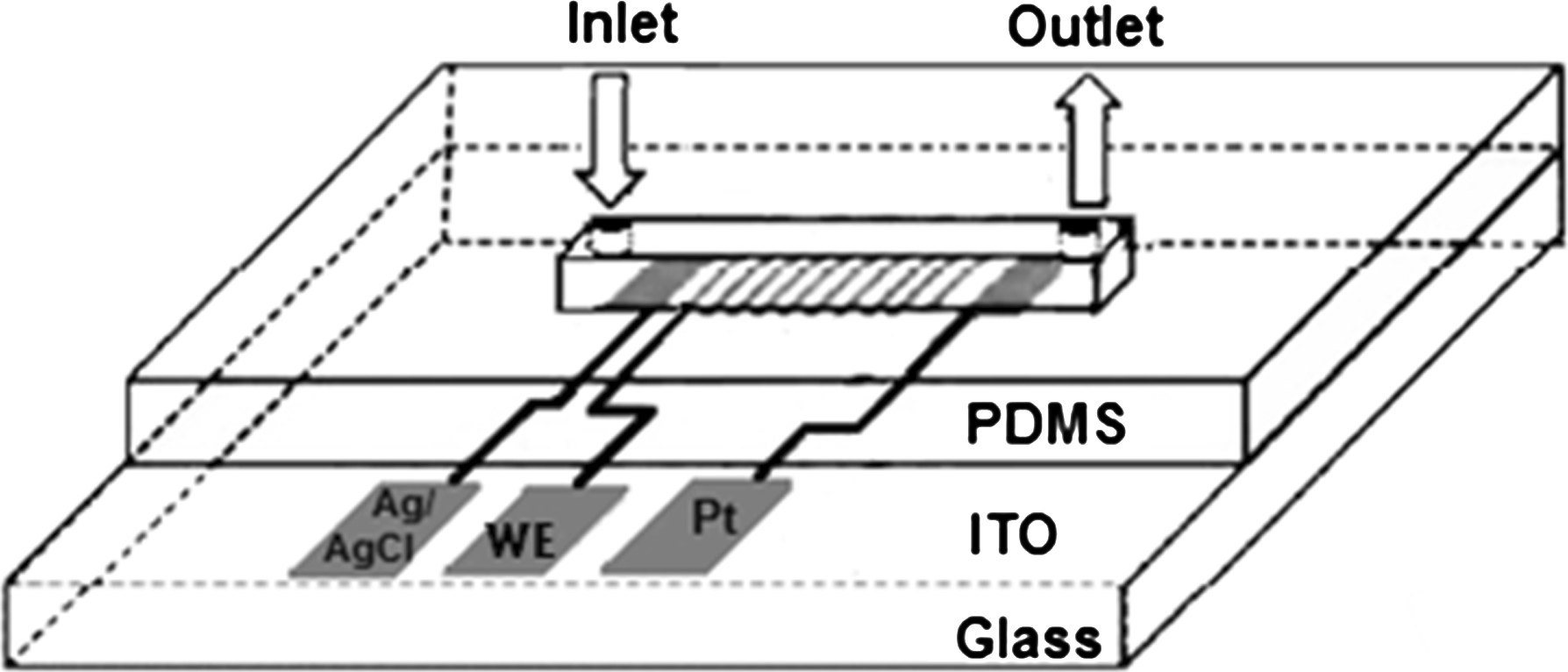

Supplement: Supplementary file 2 — Authors’ original file for figure 2 [file 40204_2012_8_MOESM2_ESM.tiff]

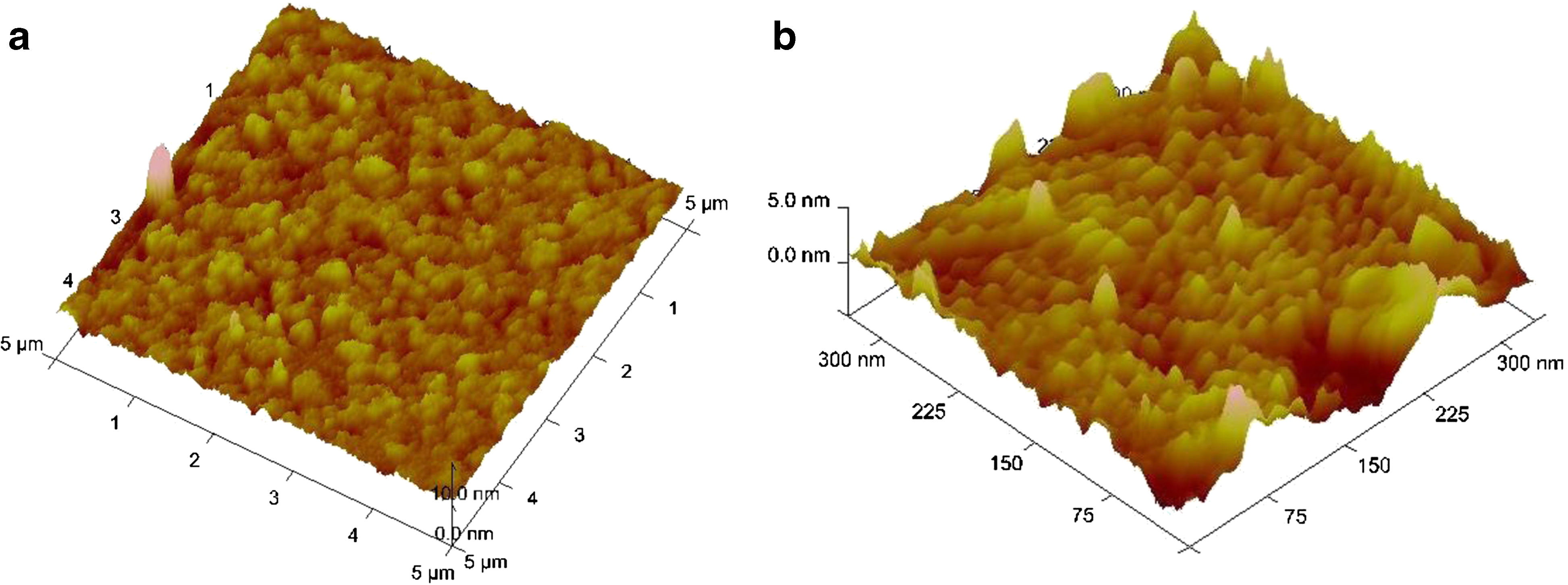

Supplement: Supplementary file 3 — Authors’ original file for figure 3 [file 40204_2012_8_MOESM3_ESM.tiff]

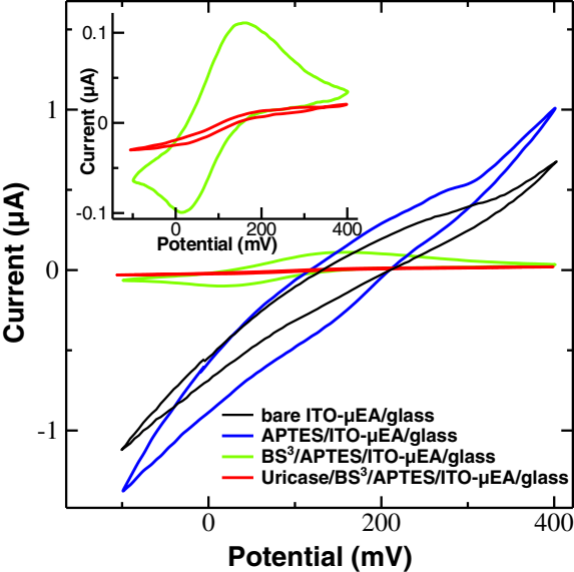

Supplement: Supplementary file 4 — Authors’ original file for figure 4 [file 40204_2012_8_MOESM4_ESM.pdf]

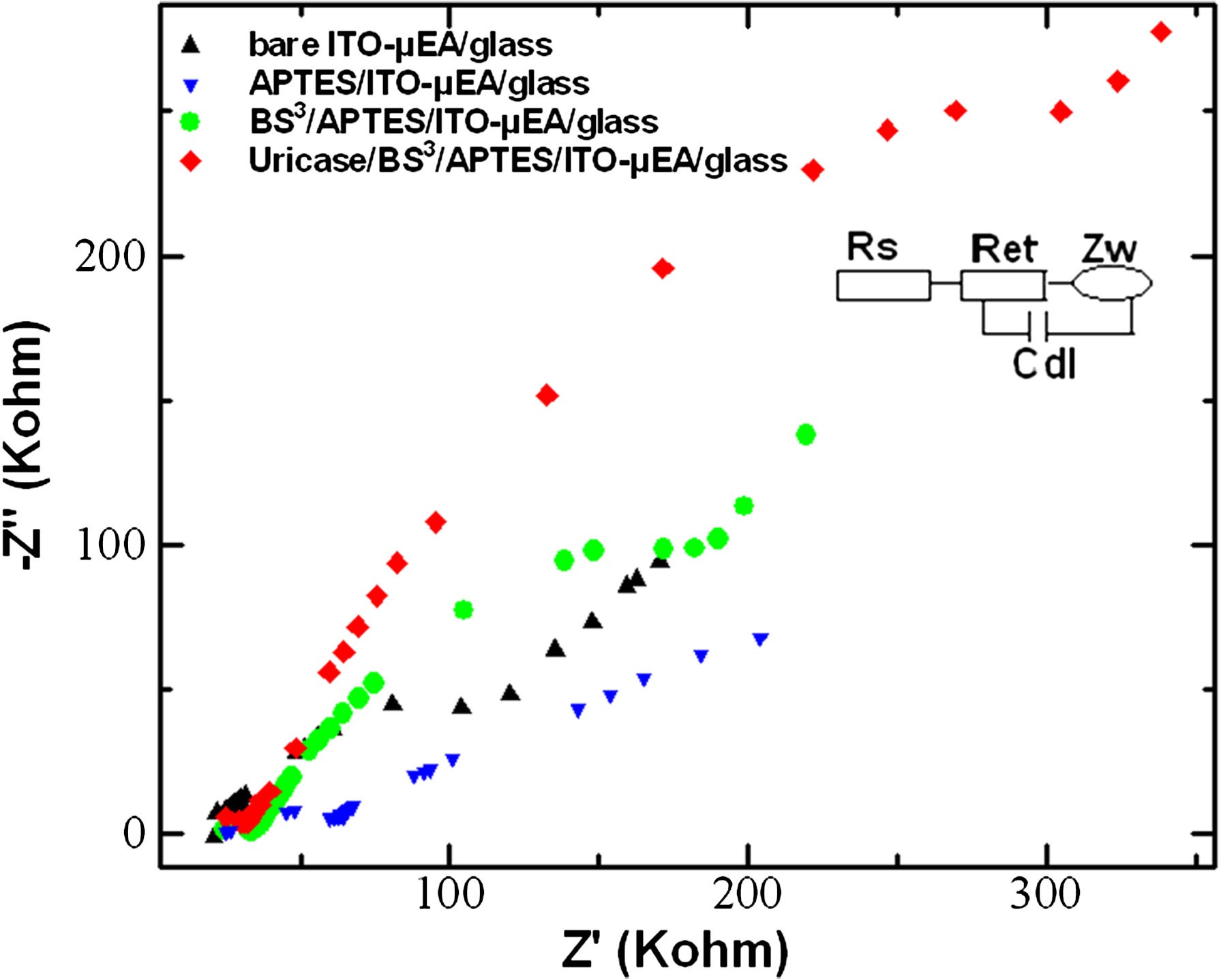

Supplement: Supplementary file 5 — Authors’ original file for figure 5 [file 40204_2012_8_MOESM5_ESM.tiff]

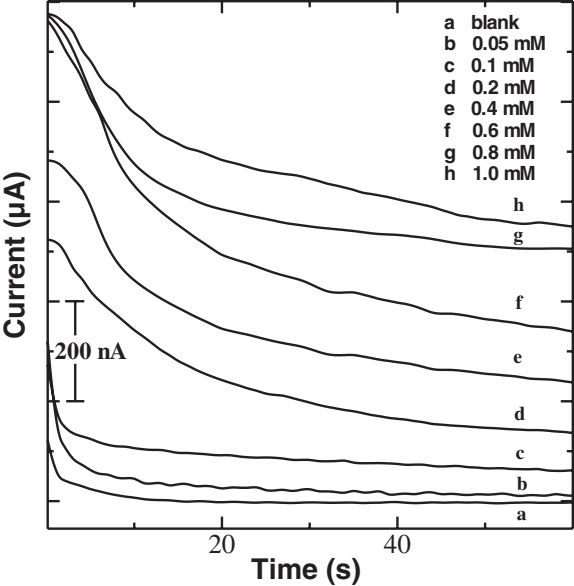

Supplement: Supplementary file 6 — Authors’ original file for figure 6 [file 40204_2012_8_MOESM6_ESM.pdf]

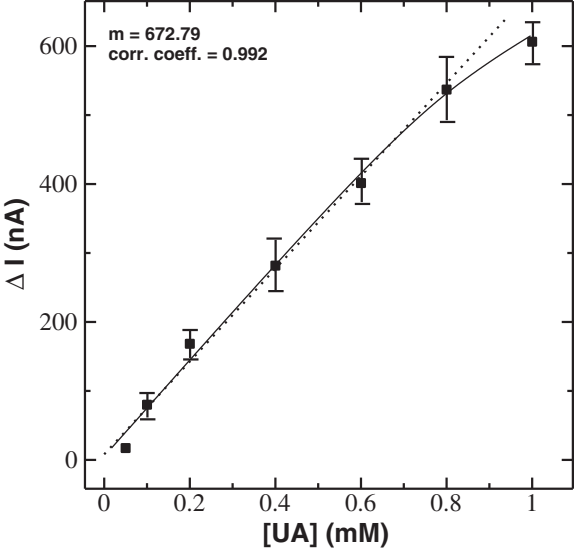

Supplement: Supplementary file 7 — Authors’ original file for figure 7 [file 40204_2012_8_MOESM7_ESM.pdf]
